# Development, inheritance and breeding potential of a recessive genic male sterile line D248A in Sesame (*Sesamum indicum* L.)

**DOI:** 10.1186/2193-1801-2-268

**Published:** 2013-06-19

**Authors:** Hongyan Liu, Minmin Yang, Kun Wu, Xinan Zhou, Yingzhong Zhao

**Affiliations:** Key Laboratory of Oil Crops Biology and Genetic Improvement, Oil Crops Research Institute, Chinese Academy of Agricultural Sciences, Wuhan, 430062 China

**Keywords:** Sesame, Genic male sterility, Recessive, Hybrid breeding

## Abstract

Genic male sterility (GMS) has great potential for heterosis exploitation in sesame (*Sesamum indicum* L.). Two spontaneous male-sterile plants were discovered in a Chinese sesame cultivar (Zhuzhi 4) in 2006. By consecutive sib mating with fertile plants from Zhuzhi 4, a new sterile line, D248A, was developed. Anatomy studies showed that D248A has thin, small and greenish anthers on which there are no or little pollen grains. The pollens are irregularly shaped and completely aborted, resulting in no germination and no formation of pollen tubes as revealed by acetocarmine stain or semi-solid suspension culture. Furthermore, D248A has a better performance in growth vigor, bloom duration and yield per plant than the other GMS lines (i.e. 95 ms-2A and 95 ms-5A). To investigate the inheritance mode of fertility, D248A was crossed and backcrossed with six varieties, and a segregating ratio of 3:1 and 1:1 for fertile and sterile plants was observed in F_2_ and BC_1_ populations, respectively. These results suggested that D248A is controlled by a recessive GMS gene. The average yield of four D248A-derived F_1_ hybrids is as high as 1695 kg·ha^-1^, which is almost twice of that of 95 ms-5A-derived F_1_ hybrids. These results indicated that this newly developed recessive GMS line has great potential in sesame hybrid breeding.

## Introduction

Sesame (*Sesamum indicum* L.) is an important and ancient oil-yielding crop with high oil quality (Bhat et al. 1999; Chung et al. 2003). Sesame seeds contain about 50–60% edible oil, which is consumed as a traditional health food for its specific antihypertensive effect and antioxidative activity (Coulman et al. 2005; Mochizuki et al. 2010; Jan et al. 2011). The global sesame production is estimated at 4 million tonnes and still steadily growing. China ranks first in sesame production (Data from FAO: 2001–2010) and sesame is a cash crop for exportation. However, the annual sesame consumption in China has substantially increased to 0.8 million tonnes in recent years, which is beyond the domestic production capacity. Therefore, more than 0.2 million tonnes of sesame were imported each year to meet domestic demand (Yang and Huang 2009). Thus, it is an urgent task for sesame breeder to further increase both yield level and total production to close the gap between demand and supply. One possible approach is to exploit heterosis in sesame by male sterility (Pal 1945; Zheng et al. 2003; Zhang et al. 2005; Zhao and Liu 2008).

Studies on male sterility in sesame can be dated back to the early 20^th^ century. First, Kumar and Abraham ( 1941) from India presented their results of comparing the differences in sterile and fertile plants in sesame variety Bijapar White. They claimed that male sterility in Bijapar White is controlled by one recessive gene, which was later reconfirmed by Kumar and Rao ( 1945). Then, Malaguti and Mazzani ( 1958) reported a partially sterile mutant in sesame, which seemed to be affected by genetics and environment as well as G×E interactions. After carefully investigating the fertility of a male sterile line in sesame, Dabral and Mandloi concluded that the male sterility is controlled by one or more recessive genes (Dabral 1968; Dabral and Mandloi 1974). By observing the fertility segregation ratio in a F_2_ population with 143 individuals, Osman and Yermanos ( 1982) concluded that the male sterility in sesame is controlled by one recessive gene, and the fertility is stable in different environments.

The first male sterile sesame germplasm in China was introduced from the United States (Tu et al. 1995). Later, several male sterile two-type lines were developed and Yuzhi 9, the first sesame hybrid in China, was bred (Zheng et al. 2003). Meanwhile, mutagenesis was also employed to generate a wide range of male sterile mutations in Oil Crop Research Institute, CAAS, from which two male sterile lines were developed and some hybrids (Zhongzhiza 1 and Zhongzhiza 2) were released (Li and Chen 1998; Zhao et al. 2008). However, the currently used male sterile lines in China were hindered by several drawbacks such as environment sensitive, incomplete sterility and the need to remove 50% male fertile plantlets from two-type line for hybrid seed production (Zheng et al. 2003). Therefore, it is of great interest to discover new source of male sterility in sesame.

The objective of this study is to develop a new GMS line from a spontaneous male sterile mutant in a Chinese sesame variety. Biometric characters such as plant height (PH), capsules per plant (CP) and seeds per capsule (SC) for this GMS line and other existing GMS lines were investigated; pollen fertility was also examined by acetocarmine or semi-solid suspension culture. A number of sib mating, selfing, and backcrossing populations segregating for fertility were generated and employed for the analysis of fertility inheritance. We hope such a study would be helpful for hybrid breeding and in long term further improving yield potential in sesame.

## Materials and methods

### Plant materials

The plant materials used in this study were male sterile line D248A (named by its field code in 2006) and its wild type cultivar Zhuzhi 4. Zhuzhi 4 was bred in Zhumadian Institute of Agricultural Science (Zhumadian, Henan, China) in 1978. Two previous GMS lines developed in our lab, 95 ms-2A and 95 ms-5A (Zhao et al. 2008), were also included for comparisons. Six varieties with different genetic background (i.e. Zhongzhi 11, Zhongzhi 12, Zhongzhi 14, Ezhi 1, Ezhi 2 and Ezhi 4) were further selected to cross with D248A to investigate its inheritance mode. All genotypes were obtained from Oil Crops Research Institute, CAAS (Wuhan, China). To evaluate the breeding potential of the new GMS line, six restorer lines (i.e. Zhu08J3, 98–4155, Hangzhi 2, 98–6204, Zhongzhi 18 and 01–2658) were used to produce F_1_ hybrids.

### Field trials and phenotyping

All plant materials were grown in summer (from 1^st^ June to 15^th^ September) in the experimental field of Oil Crops Research Institute (OCRI, CAAS), Wuhan, China (E113′53″, N29′58″), during 2006–2012. To accelerate the breeding progress, some materials were also grown in Sanya, Hainan Province, China (E109′30″, N18′12″) from January to April during 2007–2012. All plants were grown in row (2.4 m) with 0.15 m interval between individuals (resulting in approximately 16 plants per row). Rows were separated by 0.4 m space. Field management followed the normal agricultural practice.

For genetic analysis, all populations were grown in single plot with 1 to 20 rows, depending on population sizes. At flowering stage, the dates when 50% plants having their first flowers and 50% plants showing no flower were recorded for each population, from which the bloom duration (BD) was calculated. Three days before harvest, five plants were randomly sampled from the middle row in each population. After drying, PH, CP, SC and yield per plant (YP) was recorded and averaged for further comparison.

In the experiment of F_1_ hybrid evaluation, a random block design with three replicates was adopted. Each block comprised 20 rows. Before harvest, ten plants were sampled from the middle row in each block for phenotyping of PH, CP and SC. After harvest, total seed yield for each block was recorded.

### Fertility investigation

The pollen fertility of D248A was examined by two complementary methods: acetocarmine stain and pollen semi-solid suspension culture. At full-blossom stage (approximately on the 20^th^ July in Wuhan and the 5^th^ March in Sanya), anthers were collected from male sterile plant in D248A, stained by 1 g·l^-1^ acetocarmine and carefully examined by microscopy as described before (Liu 1998). The procedure of pollen grain semi-solid suspension culture was adapted from reference (Pfahler et al. 1997), with a medium containing 100 g·l^-1^ sucrose, 4 g·l^-1^ purified agar, 1 g·l^-1^ Ca(NO_3_)_2_•4H_2_O and 100 mg·l^-1^ boric acid (H_3_BO_3_). For each plant, four flowers collected from the medial part of stem were examined. A microprojector was used to determine germination percentage at ×200 magnification. Fields of 30–40 well separated grains were classified. Three counts of 100 grains each were taken on each dish after inoculation. Average germination rate was calculated across all flowers tested within the same line.

Individual plant was recorded as male fertile or male sterile based on the pollens stainability and germination. Fully developed and red stained pollen grains (with germinal pore) were classified as fertile while shriveled and unstained pollen grains (without germinal pore) were grouped as sterile.

The female fertility of D248A can be indirectly assessed by the seed set rate of hybrid produced on D248A with a fertile variety, Zhongzhi 10, as male parents.

### Genetic analysis of D248A

To investigate the inheritance of male sterility, D248A was crossed with its maintainer, D248B, to generate a sib mating population. Fertile plants in the resulting ‘D248A×D248B’ F_1_ progeny were selfed to F_2_ (plant to row). A reciprocal cross was made by using Zhongzhi 11 (a widely used variety) as female parent and D248B as male parent, which was furthered to F_2_ progeny to test the nuclear or cytoplasmic inheritance of male sterility. As female parent, D248A was also crossed with six other varieties. The resulting F_1_s were selfed or backcrossed with D248A to produce F_2_ or BC_1_ populations (as presented in Table 1).

**Table 1:** **Segregations of fertility in test crossing populations for D248A**

Generation	Population	Fertility in generation				
		Fertile plants	Sterile plants	Ratio	χ^2^value	***P*** value
F_1_	D248A×Zhongzhi 11	55	0			
	D248A×Zhongzhi 12	61	0			
	D248A×Zhongzhi 14	98	0			
	D248A×Ezhi 1	76	0			
	D248A×Ezhi 2	48	0			
	D248A×Ezhi 4	53	0			
F2	(D248A×Zhongzhi 11)	273	87	3 : 1	0.133	0.715
	(D248A×Zhongzhi 12)	187	65	3 : 1	0.085	0.771
	(D248A×Zhongzhi 14)	175	59	3 : 1	0.006	0.940
	(D248A×Ezhi 1)	206	53	3 : 1	2.843	0.092
	(D248A×Ezhi 2)	148	46	3 : 1	0.172	0.679
	(D248A×Ezhi 4)	264	102	3 : 1	1.607	0.205
BC_1_	D248A×(D248A×Zhongzhi 11)	87	68	1 : 1	2.329	0.127
	D248A×(D248A×Zhongzhi 12)	54	47	1 : 1	0.485	0.486
	D248A×(D248A×Ezhi 1)	65	71	1 : 1	0.265	0.607
	D248A×(D248A×Ezhi 2)	82	86	1 : 1	0.095	0.758
	D248A×(D248A×Ezhi 4)	54	51	1 : 1	0.086	0.770

The F_1_ hybrids as well as subsequent generations, including selfing and backcrossing populations, were studied for segregation of male sterility. The test of goodness of fit to an expected segregation ratio was performed in Microsoft Excel 2003 by calculating the probability in Chi square test. Comparison of mean difference was performed in statistic software SPSS version 9.0

## Results

### Development of male sterile line D248A

**Figure 1:**
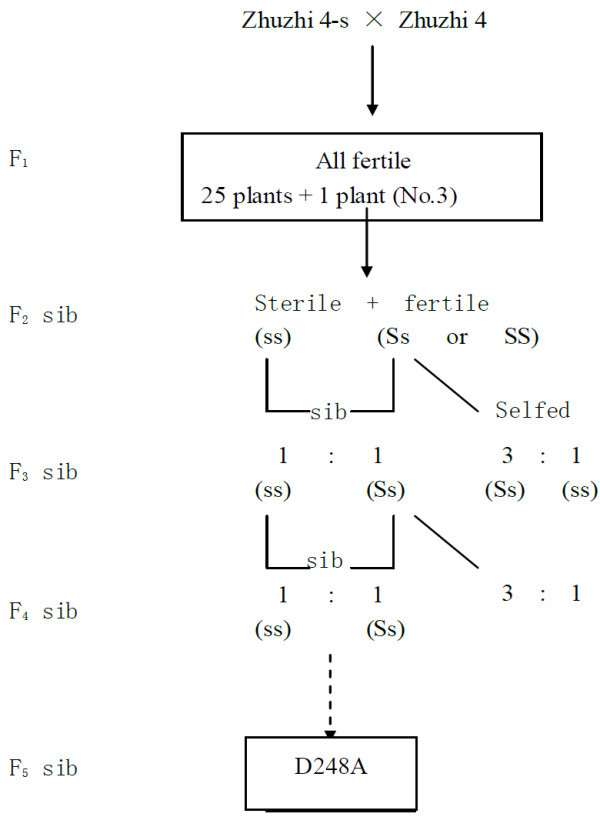
**Development and inheritance of recessive male sterile line D248A.** S, dominant fertile allele; s, recessive sterile allele.

In Zhuzhi 4, a Chinese variety released in 1978, two out of 324 plants were identified as spontaneous male sterility, with a mutation rate of 0.62%. Careful observations showed that these male sterile mutants (termed Zhuzhi 4-s) have greenish, flat and thin anthers, while its wild type parent, Zhuzhi 4, have whitish and plump ones, in which white powder can be seen after squeezing. The male sterile plants were maintained by pollinating with Zhuzhi 4, and 26 F_1_ plants were obtained, all of which were fertile. These plants were furthered to F_2_ progenies (plant to row). Male sterile plants were observed in all 26 resulting populations (in a proportion of 0.4% to 21.3%), but only one that derived from the individual F_1_ plant No.3 well fit a 3:1 ratio (22 fertile and 8 sterile plants). Then, within this F_2_ population the best male sterile plants were selected for further sib mating with fertile sister plants in the same progeny. After sib mating for five generations, a new male sterile line, D248A, was developed (Figure 1). D248A comprises approximately 50% sterile plants and 50% fertile plants. The fertile plants in this population, termed D248B, can serve as maintainer line for male sterility.

### Morphological characterization of D248A

Morphological traits such as PH, CP, SC, YP and BD were recorded for D248A and other GMS lines. Although slightly reduced PH (150 cm), less CP (76) and SC (68) were observed in D248A if compared to its wild parent Zhuzhi 4, it still had a better performance than other GMS lines (95 ms-2A and 95 ms-5A) concerning these traits (Table 2). Furthermore, BD for D248A was 10–12 d longer, which was vital for hybrid seed production. D248A also demonstrated a 79-95% higher YP than other GMS lines (Table 2), which is beneficial for yield improvement. Thus, D248A seems to have strong growth vigor and long BD, which would be of great interest for hybrid seed production.

**Table 2:** **Biometrical and floral characters of sesame male sterile lines (Wuhan, 2010)**

Line	PH (cm)	CP	SC	YP (g)	BD (d)	Anther color	Pollen	Stainability
Zhuzhi 4	152.2±5.2a	83.4±4.6a	73.7±4.3a	16.3±1.3a	47.4	White	Normal	Stained
D248A	149.7±4.4a	76.1±4.2b	68.0±4.1b	12.9±1.1b	51.3	Green	Shriveled	Unstained
95 ms-2A	132.4±3.9b	65.2±3.8c	56.6±3.5c	6.6±0.7c	41.2	Brown	Shriveled	Unstained
95 ms-5A	112.3±3.7c	69.8±4.6c	60.4±3.9c	7.2±0.9c	38.8	Brown	Shriveled	Unstained

Note: PH, plant height; CP, capsules per plant; SC, seeds per capsule; YP, seed yield per plant; BD, bloom duration. Value was presented as mean ± S.E. The same letter represents no difference at *P*<0.05.

### Male and female fertility of D248A

**Figure 2:**
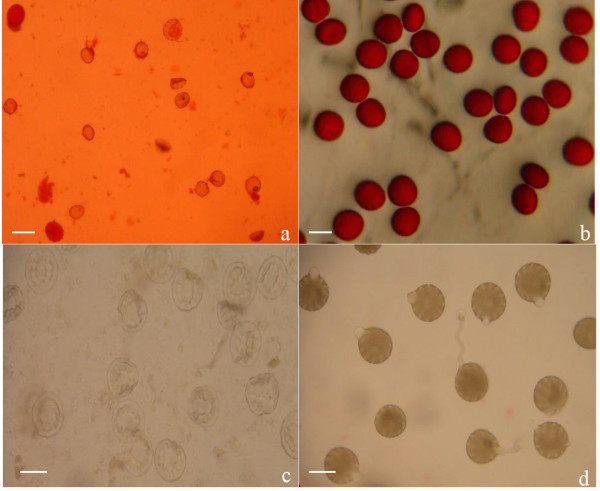
**Pollen fertility of male sterile line D248A examined by acetocarmine (a and b) and suspension culture (c and d).** (**a** and **c**: Pollens from D248A; **b** and **d**: Pollens from Zhuzhi 4). Bars=10 μm.

D248A is characterized by thin, flat and green anther. Generally, the pollen grain of D248A is small, unstained (Table 2) and irregular in shape (triangular or rough), inside which the cytoplasm is unevenly distributed, with little or no contents (Figure 2a). As for some pollen grains, there are no or little nuclei inside, or the contents had already spilled (Figure 2a). In contrast, pollen grains from fertile plants of Zhuzhi 4 are larger, fully developed into round or oval shaped, and deeply stained (Figure 2b).

The fertility of pollen grains was also investigated by semi-solid suspension culture for 30 minutes. In this examination, pollens from D248A were found to be crinkled, in plasmolysis nature, and there were no contents inside. Such pollens could not germinate and form pollen-tubes (Figure 2c). However, the pollen grains of fertile plant from Zhuzhi 4 have visible germinal furrows and germinal pores (Figure 2d; 11–15 germinal furrows per grain), with a germination rate > 90% as examined by microscope.

The female fertility of D248A can be indirectly evaluated by comparing the capsule set with that of its sister plant (D248B). For this purpose, we manually pollinated D248A or D248B as female parents with the same fertile cultivar Zhongzhi 10 as male parent. The capsule set of ‘D248A×Zhongzhi 10’ F_1_ was 94.8%, which was very close to that of ‘D248A×Zhongzhi 10’ F_1_ (98.5%), indicating that D248A is intact in term of female fertility (stigma).

### Inheritance of D248A

A number of plant-wise sib mating of D248A with D248B and selfing of pollinator plants were carried out in 2010. Six out of 7 sib mating populations showed a segregation of fertile to sterile plants in a 1:1 ratio. Also, all the seven selfed progenies of the respective pollinators (sister plants) showed a segregation at 3:1 (fertile/sterile) ratio. Thus, it was inferred that only one gene locus is responsible for the male sterility.

To determine whether the male sterile gene is in the cytoplasm or nuclear, a reciprocal cross i.e. ‘Zhongzhi 11 × D248B’ was made. Thirteen F_1_ plants were obtained and all are fertile, which were then selfed to generate 13 F_2_ populations (plant to row). Among these populations, seven are segregating for a 3:1 ratio, while the rest six do not segregate at all. Thus, approximately 50% of F_2_ populations showed fertility segregation, which is expected for one gene model. This observation indicated that the fertile maintainer line, D248B, also carries the male sterile gene, which is resident in the nuclear (Figure 1).

To determine whether the male sterility gene (*s*) is in dominant or recessive inheritance, D248A was crossed with six other varieties as male parents. All F_1_ plants are completely fertile (Table 1), suggesting that the *s* gene is recessive. The F_1_ plants were advanced to F_2_ by selfing and to BC_1_ by backcrossing with D248A to confirm its inheritance. In F_2_ progenies, the fertility segregation ratio is not significantly different from 3:1. In 13 BC_1_ progenies obtained, 12 are segregated in a 1:1 ratio and only 1 is deviated from 1:1 possibly due to small population size (Table 1). These results further confirmed that the gene responsible for male sterility in D248A is recessive.

Together, the above investigations suggested that the sterility trait in D248A, which is stable in various genetic backgrounds, is controlled by a single recessive nuclear gene, and can be passed on to offspring by both sterile parent (D248A) or fertile parent (D248B).

### Yield performance of D248A-derive F_1_ Hybrid

To evaluate the potential utilization of D248A in hybrid breeding, four F_1_ hybrids were generated and compared with four other hybrids derived from 95 ms-5A. Seed yield and component traits were measured, as presented in Table 3. The seed yields of D248A derived hybrids range from 1181 kg·ha^-1^ to 1952 kg·ha^-1^, with an average of 1695 kg·ha^-1^, which is 110% higher than Zhongzhi 14 (an open-pollinated cultivar widely used in China), the CK. The yield of 95 ms-5A derived hybrids, however, is only 10% higher than CK, with an average of 885 kg·ha^-1^. Thus, the average yield of D248A derived hybrids is almost twice of 95 ms-5A derived hybrids, indicating that D248A has great potential in hybrid breeding. As for yield component traits such as PH, CP, SC and seed weight, D248A-derived hybrids also showed a moderate heterosis.

**Table 3:** **Comparison of yield components in F**_**1**_**hybrid (Wuhan, 2012)**

F_1_hybrid	PH (cm)	CP	SC	1000-seed weight (g)	Seed yield (kg·ha^-1^)	% yield increase
D248A×Zhu08J3	175.8±4.5	160.9±6.2	73.9±1.3	3.60±0.08	1952.5±155.2a	142.4
D248A×98-4155	165.2±5.3	152.4±4.5	74.6±2.1	3.45±0.11	1863.2±123.5a	131.3
D248A×Hangzhi 2	184.6±5.9	142.7±4.8	75.4±1.9	3.59±0.07	1783.7±89.9a	121.5
D248A×98-6204	163.0±7.4	129.6±3.6	76.5±3.4	3.14±0.13	1180.8±190.7b	46.6
95 ms-5A×Zhongzhi 18	172.9±5.6	127.6±2.8	67.7±2.1	3.29±0.13	1170.0±140.1b	45.3
95 ms-5×01-2658	125.2±3.8	113.8±3.4	73.4±2.8	3.11±0.21	941.5±40.5c	16.9
Zhongzhi 14 (CK)	135.2±3.9	120.1±3.9	69.0±1.5	3.47±0.17	805.3±59.6 cd	0.0
95 ms-5×Zhongzhi 11	116.2±3.1	98.4±2.7	67.5±2.5	3.30±0.21	716.7±67.6d	−11.0
95 ms-5×98-6204	122.4±2.8	81.3±2.3	66.7±1.4	3.50±0.18	712.4±88.7d	−11.5

Note: Abbreviations are the same in Table 2.% yield increase= (hybrid yield – CK yield)*CK yield^-1^*100.

## Discussion

In sesame, both GMS and cytoplasmic male sterility (CMS) systems have been identified (Kumar and Abraham 1941; Osman and Yermanos 1982; Tu et al. 1995; Anitha and Ganesan 2000; Kavitha et al. 2000; Wang and Wang et al. 2002). Among them, some are unstable in male sterility expression. For example, the sib-mated progenies derived from a chemical induced male sterile mutant produce a lower frequency of male sterile plants than the expected proportion of 50%, indicating that the induced GMS has not attained stability (Anitha and Ganesan 2000). In the present study, we have developed a new GMS line which is complete (Table 2; Figure 2) and stable across different genetic backgrounds even different environments (Wuhan and Sanya, China), which is similar to a previous report by Kavitha et al. ( 2000) who found that the expression of pollen sterility is stable in progenies of backcross generation (BC_11_F_1_) at two different locations and that it exhibits male sterility (>98%) even at high day temperature (36°C).

Although a number of male sterility lines have been reported, most of them cannot be used in breeding practice mainly due to incomplete male sterility, poor agronomic traits, or poor general combining ability (GCA). For instance, Anitha and Ganesan ( 2000) obtained three chemical-induced completely sterile plants, with no or sterile pollen. However, these plants show general reduction in the values of all biometrical characters compared to wild parent. The new sterile line developed in present study, although also being classified as recessive GMS, has a number of advantages when compared to previous ones. Being developed from a spontaneous mutant in Zhuzhi 4, a widely used cultivar in China, D248A has very good agronomic performance such as growth vigor, long BD (Table 2) and high GCA (unpublished data), which can be directly used in breeding practice. Currently, four F_1_ hybrids with 47-142% yield advantage over check variety have been developed from D248A (Table 3), which will be further tested in National Regional Trail.

GMS has great potential for hybrid seed production for several crops such as rice (Borkakati and Virmani 1996), wheat (Liu et al. 1997), cotton (Jyotiba et al. 2010) and rapeseed (Chen et al. 1993). The obvious merits of recessive GMS include the completeness of sterility, no potential adverse cytoplasmic effect and the availability of large number of restorer lines. These merits insure more chances of developing super F_1_ hybrid and less risk of producing impure hybrid seeds. However, like previous GMS lines, D248A also requires the removal of 50% fertile plants for hybrid production, which would be laborious and cost inefficient. To mitigate this situation, several approaches including pleiotropism and nonallelic interaction have been proposed (Rao et al. 1990). The most promising approach, however, is from rapeseed (*Brassica napus* L.). Chen et al. ( 1993) reported a new recessive GMS line (9012A) in which the sterility is conditioned by two recessive genes and an epistatic suppression gene (*esp*). Later, they successfully developed it into a ‘three-line’ system (i.e. sterile line, temporary maintainer line and restorer line), which is as effective as traditional CMS system for massive hybrid seed production (Chen and Hu 1998,2003). In our study, the possibility of a similar *esp* gene in sesame cannot be ruled out. In fact, Wang and Wang ( 2002) have already shown some evidences for this possibility. In their study, the male sterility of sesame is controlled by 1–2 recessive genes plus an epistatic gene. Screening sesame germplasm to identify such a gene is now underway in our lab. Much more efforts should be made on developing D248A into a ‘three-line system’ in the future.
